# The joint associations of smoking and obesity with subsequent short and long sickness absence: a five year follow-up study with register-linkage

**DOI:** 10.1186/s12889-017-4997-x

**Published:** 2017-12-28

**Authors:** Eira Roos, Tea Lallukka, Eero Lahelma, Ossi Rahkonen

**Affiliations:** 1 Department of Public Health, University of Helsinki, Post Box 20, 00014 Helsinki, Finland; 2 Finnish Institute of Occupational Health, Helsinki, Finland

**Keywords:** Absenteeism, Cohort, Epidemiology, Life-style, Middle aged, Mid-life, Work disability

## Abstract

**Background:**

Both smoking and obesity are separately associated with sickness absence. Unhealthy lifestyle habits and health conditions may occur concurrently yet studies focusing on their joint association are few. This study examined the joint associations of smoking and obesity with sickness absence (SA).

**Methods:**

A mail survey among employees of the City of Helsinki, Finland, during 2000–2002 included data on obesity, smoking and covariates (*N* = 8960, response rate 67%, 80% women). These data were prospectively linked with register data on self- (1–3 days) and medically certified (4 days or longer) SA among those consenting to the linkage (*n* = 6986). Pregnant, underweight and those with missing data on key variables were excluded (*n* = 138). The total number of participants included in the analyses was 6847. The follow-up time was 5 years. Poisson regression was used to calculate rate ratios (RR).

**Results:**

Among women and men smoking and obesity were associated with self-certified SA. Among women there was a joint association with self-certified SA (obese smokers RR 1.81, 95% CI 1.59–2.07).

Among women and men smoking and obesity were jointly associated with medically certified SA (for obese smoking women RR 2.23, 95% CI 1.93–2.57, for obese smoking men RR 2.69, 95% CI 2.03–3.55). Associations remained after adjustments for socioeconomic position, working conditions, health behaviours and self-rated health.

**Conclusion:**

Both smoking and obesity are jointly associated with all lengths of sickness absence. Support measures for smoking cessation and prevention of obesity could likely to reduce SA.

## Background

Health conditions and health behaviours, but also working conditions, socio-economic position and gender all influence sickness absence [1, 2].

Regarding health conditions and health behaviours, smoking and obesity are often regarded as some of the strongest risk factors for sickness absence [3–8]. In a Finnish study among municipal kitchen workers both obesity and smoking predicted future sickness absence due to musculoskeletal pain [3]. A similar result was gained in another Finnish study among municipal employees regarding sickness absence due to any cause [4]. Also an Italian study found that obesity and smoking were associated with later sickness absence [5]. Smoking and obesity have been identified as risk factors for sickness absence also in a Danish study among female health care workers [6], in a Dutch study among construction workers [7] and in an American study among 3790 employees [8]. Despite the abundance of studies uncertainties still remain as some of these studies have examined sickness absence due to specific symptoms only [3] or among specific occupations [3, 6, 7]. As sickness absence is strongly associated with socio-economic position [9, 10] these studies do not necessarily allow generalization to general work force.

It is possible that risk factors such as obesity and smoking bundle together [11], nevertheless we lack studies focusing on the joint association of smoking and obesity with sickness absence. Our recent study showed that there is a joint association of smoking and obesity with premature death, such that obesity intensifies the effect of smoking on mortality risk [12]. If such an effect were present also for sickness absence, it would imply that work disability prevention measures should be targeted to counteract the increased risk of sickness absence among obese smokers in order to enhance their well-being and work ability.

Most of the previous research on sickness absence has assessed the risk of long term sickness absence as it denotes serious health problems and the risk of disability retirement, while short sickness absence spells, especially self-certified, have been much less studied [4]. The reasons behind this are perhaps the lack of reliable data for self-certified sickness absence but also a common misconception that short sickness absence is insignificant as it is typically associated with seasonal flus, common colds and gastroenteritis, i.e. conditions that anyone is prone to catch. However, short sickness absence, may denote the first signs of emerging health problems and work disability [13] and lead to longer sickness absence spells [14]. In clinical practice the high rates of short sickness absence are commonly seen among heavy users of health services. In addition, the recent economic turmoil in Europe has pressured employers to streamline work procedures to high efficacy, meaning that even short sickness absence spells can cause major problems in work community and organization.

The aim of this study was to examine the joint associations of smoking and obesity with short self-certified and longer, medically certified sickness absence in a multi-occupational employee cohort, taking into account the contribution of covariates that have been shown to associate with smoking, obesity and sickness absence.

## Methods

This study is part of the ongoing Helsinki Health Study on midlife employees of the City of Helsinki, Finland. Baseline mail questionnaire surveys were conducted among all employees turning 40, 45, 50, 55 and 60 years during years 2000–2002 (*n* = 8960, response rate 67%, 80% women) [15]. The survey included questions on health, health behaviours, weight, height, working conditions and sociodemographics. Non-response analyses have shown that the data represent the target population satisfactorily although men, manual workers and younger employees were somewhat underrepresented among the respondents [15, 16]. The respondents represent several hundreds of different manual and non-manual occupations.

The survey data were linked prospectively with employer’s register-based sickness absence data for those that gave consent to such linkage (78%). Analyses of non-consenting have shown that the consenters represent the target population satisfactorily [16].

### Variables

#### Independent variables

The baseline questionnaire included questions on current smoking. Cigarette, cigar and pipe smoking was asked and 99% of the smokers were cigarette smokers. According to the responses, smoking was categorized into non-smokers and current smokers. A small number of people with missing data on smoking were excluded (*n* = 46) from the analyses.

**Fig. 1:**
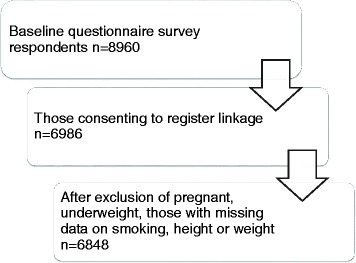
Flow chart of the study population

Self-reported weight and height were derived from the baseline questionnaire and body mass index (BMI) was calculated according to the standard formula, i.e. weight in kilograms divided by squared height in meters. Weight was categorized as non-obese or obese, using BMI 30 (kg/m^2^) as a cut-off value. Underweight (BMI < 18.5 kg/m^2^) respondents (*n* = 22), pregnant respondents (*n* = 14) and those with missing data on weight or height (*n* = 56) were excluded. Underweight respondents were excluded as though it is known that their risk for sickness absence is heightened [6, 17]. Their small number made reliable analyses of their risk for sickness absence not feasible. Total number included in the analyses was 6847. Flow chart of the study population is presented in the Fig. 1.

The joint variable of smoking and weight was then formed by cross-tabulating smoking and obesity yielding four categories: non-obese non-smokers, non-obese smokers, obese non-smokers, and obese smokers. Non-obese non-smokers were used as the reference category in the analyses.

#### The dependent variables

The outcome of the study was the number of sickness absence spells during the five-year follow up after responding to the baseline questionnaire. Data on sickness absence spells were based on the employer’s personnel register. These registers are based on employees’ salaries and can thus be considered accurate. Sickness absence spells were divided into short self-certified (1–3 days) and long (4+ days) medically certified sickness absence spells. The employees of the City of Helsinki can take self-certified sickness absence lasting 1–3 days and after three days absence medical certification is required.

#### Covariates

Covariates were age, socio-economic position, working conditions, alcohol consumption, leisure-time physical activity and self-rated health. Covariates were chosen as previous studies have shown that they are associated with determinants and the outcome of the study [18–23]. Covariates were obtained from the baseline questionnaire, except for occupational class, which was obtained from personnel register.

Age was treated as a continuous variable. Occupational classes were manual workers, routine non-manual employees, semi-professionals or managers and professionals. Managers and professionals were considered as a reference group as their risk for sickness absence is typically lowest [24]. Working conditions included work arrangements, physical working conditions, and psychosocial working conditions. Work arrangements were encoded as a dichotomous covariate: employees doing regular daytime work formed one group, and the rest formed another group. Regular daytime work was considered as a reference group. Physical working conditions were assessed with an 18-item questionnaire developed at the Finnish Institute of Occupational Health [25]. Factor analyses of the questionnaire provided the following factors: (i) physical workload, such as uncomfortable postures, or heavy physical exertion; (ii) hazardous exposures, such as dirt, solvents, or mold; and (iii) computer work, including sedentary work. Each factor score was divided into quartiles and the lowest quartiles were used as reference groups. Karasek’s job content questionnaire was used to measure psychosocial working factors [26]. Separate summary scores for job demands (9 items) and job control (9 items) were divided into quartiles and the lowest quartiles served as reference groups. Alcohol consumption was asked as weekly consumption of spirits, wine and beer and each unit was converted to 12 g units of pure alcohol. The consumption was divided into quartiles. The lowest quartile was used as a reference group as heavy alcohol use is associated with sickness absence [21]. Leisure time physical activity was measured as average weekly hours of leisure-time physical activity and converted to metabolic equivalent (MET) hours [27] according to the reported intensity. The MET-hours were then divided into quartiles and the highest quartile was used as a reference group as high exercise levels are associated with less sickness absence [22]. Self-rated health was assessed with a single question derived from the Short Form 36 Health questionnaire (SF-36) [28]. Good health was used as a reference group as poor self-rated health is as a strong and independent predictor of work disability [23] and mortality [29, 30].

### Statistical methods

First, the number of sickness absence spells and the amount of sickness absence days per hundred person years were calculated. Next, Poisson regression models were used to examine the joint association of smoking and obesity with short and long sickness absence. Men and women were analysed separately as statistically significant interaction (*p*-value = 0.0005) was observed between gender and the joint obesity and smoking variable in relation to sickness absence. Four different models were analysed. First model was adjusted for age, the second one was additionally adjusted for occupational class and working conditions. The third model was adjusted for age, alcohol consumption and leisure-time physical activity and the last model was adjusted for age and self-rated health. As adjustments had but a marginal effect on the results, only the first model (Model 1) adjusting for age and the full model (Model 2) adjusting for all covariates are shown.

SAS 9.3 was used in the analyses of the data.

#### Ethical considerations

The Helsinki Health Study protocol has been approved by the ethics committees of the Department of Public Health, University of Helsinki, Finland and the City of Helsinki Health Authorities.

## Results

Table 1 shows the descriptive characteristics of key variables of the study population. Sickness absence spells per hundred person years and sickness absence days per person years by obesity and smoking are shown in the Table 2. Non-obese non-smokers had the lowest amount of short and long sickness absence spells as well as sickness absence days both among women and men.

**Table 1:** Key characteristics of the study population at baseline

Variables	Women (*n* = 5388)	Men (*n* = 1459)
Age in years (SD)	50.2 (6.6)	49.3 (6.6)
Body Mass Index, BMI (SD)	25.5 (4.4)	26.5 (3.9)
Smoking	22%	26%
Socio-economic position		
Professionals and managers %	28%	44%
Semiprofessionals %	19%	20%
Routine non-manual %	42%	10%
Manual workers %	12%	27%
Regular day-time work	79%	70%
Poor self-rated health	25%	28%
MET^a^ hours/week (SD)	28 (22)	33 (29)
Alcohol units/week (SD)	4.0 (5.1)	9.2 (10)

^a^metabolic equivalent value

**Table 2:** Sickness absence spells per one hundred person years (100 py) and sickness absence days per person year by baseline body weight and smoking

	Women				Men			
	N	Short n/100 py	Long n/100 py	days/py	N	Short n/100py	Long n/100py	days/py
Non-obese non-smoking	3544	133	66	14	914	72	41	10
Non-obese smoking	1059	184	104	23	322	136	63	15
Obese non-smoking	617	182	117	28	155	89	67	17
Obese smoking	168	243	144	32	68	126	110	32
All	5388	152	82	18	1459	90	51	13

Poisson regression models (Table 3) showed that among women both smoking (non-obese smokers RR 1.34, 95% CI 1.26–1.44) and obesity (obese non-smokers RR 1.40, 95% CI 1.28–1.52) were associated with self-certified sickness absence. They were also jointly associated with self-certified sickness absence (obese smokers RR 1.79, 95% CI 1.56–2.04). Among men smoking was strongly associated with self-certified sickness absence (non-obese smokers RR 1.81, 95% CI 1.55–2.12) whereas the association between obesity and self-certified sickness absence was weaker (obese non-smokers RR 1.28, 95% CI 1.00–1.63). Obesity did not strengthen the association between smoking and self-certified sickness absence (obese smokers RR 1.80, 95% CI 1.33–2.45). Adjusting for socioeconomic position, working conditions, leisure-time physical activity, alcohol consumption, and self-rated health attenuated the risk only modestly.

**Table 3:** The joint association of smoking and obesity with self-certified sickness absence among women (*n* = 5388) and men (*n* = 1459), showing rate ratios (RR) and their 95% confidence intervals (95% CI)

	Non-obese non-smoking	Non-obese smoking		Obese non-smoking		Obese smoking	
	RR	RR	95% CI	RR	95% CI	RR	95% CI
Women							
Model 1	1.00	1.34	1.26–1.44	1.40	1.28–1.52	1.79	1.56–2.04
Model 2	1.00	1.25	1.17–1.33	1.28	1.18–1.40	1.58	1.39–1.80
Men							
Model 1	1.00	1.81	1.55–2.12	1.28	1.00–1.63	1.80	1.33–2.45
Model 2	1.00	1.56	1.32–1.84	1.09	0.86–1.39	1.56	1.14–2.12

Model 1 is adjusted for age

Model 2 is adjusted for age, socio-economic position, working conditions, leisure-time physical activity, alcohol consumption and self-rated health

When considering medically certified sickness absence among women both smoking (non-obese smokers RR 1.56, 95% CI 1.44–1.68) and obesity (obese non-smokers RR 1.73, 95% CI 1.58–1.89) were associated with sickness absence, and again there was also a joint association (obese smokers RR 2.23, 95% CI 1.93–2.57) (Table 4). Among men both smoking (non-obese smokers RR 1.57, 95% CI 1.31–1.88) and obesity (obese non-smokers RR 1.71, 95% CI 1.35–2.16) were associated with medically certified sickness absence, and their joint association was particularly strong (obese smokers RR 2.69, 95% CI 2.03–3.55). Again, adjustments for socioeconomic position, working conditions, leisure-time physical activity, alcohol consumption, and self-rated health attenuated the risk somewhat but it remained.

**Table 4:** The joint association of smoking and obesity with medically certified sickness absence among women (*n* = 5388) and men (*n* = 1459), showing rate ratios (RR) and their 95% confidence intervals (95% CI)

	Non-obesenon-smoking	Non-obese smoking		Obese non-smoking		Obese smoking	
	RR	RR	95% CI	RR	95% CI	RR	95% CI
Women							
Model 1	1.00	1.56	1.44–1.68	1.73	1.58–1.89	2.23	1.93–2.57
Model 2	1.00	1.32	1.23–1.41	1.40	1.29–1.52	1.71	1.50–1.94
Men							
Model 1	1.00	1.57	1.31–1.88	1.71	1.35–2.16	2.69	2.03–3.55
Model 2	1.00	1.20	1.02–1.42	1.24	1.00–1.53	1.96	1.52–2.52

Model 1 is adjusted for age

Model 2 is adjusted for age, socio-economic position, working conditions, leisure-time physical activity, alcohol consumption and self-rated health

## Discussion

This study examined the joint association of smoking and obesity with sickness absence, considering both short, self-certified sickness absence and longer, medically certified sickness absence among midlife employees. While both smoking and obesity increased the risk of both self- and medically certified sickness absence, the main finding was that smoking and obesity were jointly associated with sickness absence.

In previous research both smoking and obesity have been identified as separate risk factors for long spells of sickness absence [3–8]. Our results show that among women smoking and obesity are jointly associated, meaning that obese smokers have particularly high risk of both self- and medically certified sickness absence. Among men this joint association appears only when considering medically certified sickness absence, however, the joint association is even stronger than among women, referring to multiplicative effect. It is known that smoking is associated with increased risk of many medical conditions that can cause either temporary or more permanent work disability. Such conditions include musculoskeletal disorders [31], respiratory problems [32] as well as heart diseases [33], all of which are also associated with obesity [34]. The mechanism of this interaction is not known and requires further studies.

Only one previous study, using the same data as the present study has examined short self-certified sickness absence and found that smoking and obesity are separately associated with increased risk of short sickness absence [4]. However, their joint associations with sickness absence was not examined in the previous study. The present study extends these results by showing that among men smoking is more strongly associated with short self-certified sickness absence than obesity whereas among women smoking and obesity are jointly associated even with short sickness absence. Short, self-certified sickness absence and long sickness absence denote different health problems in the same spectrum of work disability and thus self-certified sickness absence can be considered as a first marker of possible problems in employee’s health or coping [13].

Adjusting for health behaviours, socio-economic position, working conditions and self-rated health attenuated the estimates only modestly. In sensitivity analyses self-rated health had the strongest effect, whereas adjusting for other health behaviours had but negligible effects on the estimates. Self-rated health [23], as well as self-assessments of work disability [34] have been shown to predict later work disability and thus its contribution to our results is expected. Although health behaviours had negligible effects in our study, physical activity or alcohol use are significant risk factors for sickness absence [21, 22]. Thus our results suggest that obesity and smoking are independent and joint risk factors.

This study did not examine the diagnoses leading to sickness absence. However, it is known that obesity and smoking are associated with many diseases that may lead to various lengths of sickness absence. Such diseases include for example lung conditions such as asthma, Chronic Obstructive Pulmonary Disease (COPD) and lung cancer, musculoskeletal disorders such as back pain and as well as cardiovascular diseases [33, 35–37]. As the mechanisms that lead to mentioned diseases are assumedly different for smoking and obesity it is plausible that their joint association with these diseases is additive.

Intentional weight loss or quitting smoking was not examined in this study. Therefore conclusions on their associations on the risk of sickness absence cannot be drawn. However, our results suggest that quitting smoking or decreasing relative weight could potentially reduce sickness absence. Changing health behaviours is, however, challenging and often requires support measures from health professionals. It is known that quitting smoking [38] appears to be more successful than weight reduction [39]. In occupational health care context it might be more cost effective to focus on smoking cessation support when both risk factors are present although obesity itself is a strong risk factor of work disability [40].

The strengths of this study include a large multi-occupational cohort and register-based data on sickness absence as well as the possibility to include short self-certified sickness absence. It has been previously reported that participants of this study represent the socioeconomic spectrum satisfactorily, although younger and manual employees were slightly less likely to participate at baseline [15, 16]. The City of Helsinki is the largest employer in Finland with nearly 40,000 employees, representing hundreds of different occupations in both blue- and white-collar jobs. However, it should be noted, that the social security systems and the compensation provided for sickness absence vary from country to country and as these factors influence how prone an employee is to take a sick leave these results are not necessarily generalizable to populations that are under different social security system. The survey data relies on self-reports and although self-reported weight has been shown to correspond well with measured weight [41], there might be reporting bias on smoking and other health behaviours. Sensitivity analyses showed that non-obese smokers gave consent to linkage to sickness absence registers slightly less often (81%) than the other groups (83–88%) which suggests that our results are likely somewhat conservative. According to previously published analyses, those consenting to register linkage represent target population satisfactorily [16]. Due to the small number of cases we were unable to examine separately never-smokers and previous smokers.

## Conclusion

Both smoking and obesity are jointly associated with all lengths of sickness absence. However, to fully understand their joint associations further research is required. Support measures for smoking cessation and prevention of obesity are suggested especially in the occupational health setting.

## Abbreviations

BMI: Body Mass Index
CI: Confidence interval
MET: Metabolic Equivalent
PY: Person years
RR: Rate ratios
SA: Sickness absence
